# Predicting MicroRNA Biomarkers for Cancer Using Phylogenetic Tree and Microarray Analysis

**DOI:** 10.3390/ijms17050773

**Published:** 2016-05-19

**Authors:** Hsiuying Wang

**Affiliations:** Institute of Statistics, National Chiao Tung University, Hsinchu 30010, Taiwan; wang@stat.nctu.edu.tw or hsiuyingwang@nctu.edu.tw; Tel.: +886-3571-2121 (ext. 56813); Fax: +886-3572-8745

**Keywords:** cancer, microarray, microRNA, sequence, phylogenetic tree

## Abstract

MicroRNAs (miRNAs) are shown to be involved in the initiation and progression of cancers in the literature, and the expression of miRNAs is used as an important cancer prognostic tool. The aim of this study is to predict high-confidence miRNA biomarkers for cancer. We adopt a method that combines miRNA phylogenetic structure and miRNA microarray data analysis to discover high-confidence miRNA biomarkers for colon, prostate, pancreatic, lung, breast, bladder and kidney cancers. There are 53 miRNAs selected through this method that either have potential to involve a single cancer’s development or to involve several cancers’ development. These miRNAs can be used as high-confidence miRNA biomarkers of these seven investigated cancers for further experiment validation. miR-17, miR-20, miR-106a, miR-106b, miR-92, miR-25, miR-16, miR-195 and miR-143 are selected to involve a single cancer’s development in these seven cancers. They have the potential to be useful miRNA biomarkers when the result can be confirmed by experiments.

## 1. Introduction

MicroRNA (miRNA) is a short non-coding RNA around 22 nt, which suppresses gene expressions via translational suppression or involves mRNA degradation by binding to 3′-untranslated regions (3′UTR). It has been estimated that miRNAs regulate about 30% of human genes [1]. The first miRNA was discovered in 1993, which downregulates the levels of lin-14 during the development of *C. elegans* [2]. Previous studies indicated that miRNAs play a key role in biological processes, such as development, cell proliferation and cell death. Thus, altered miRNA expression is likely to contribute to human diseases [3]. As a result, miRNA expression may be a useful tool for disease detection [4].

Calin *et al.* [5] first linked miR-15 and miR-16 to cancer in 2002. After that, many studies have shown that the expression of miRNAs is altered in many cancer samples, and they can initiate carcinogenesis or drive progression [6]. In addition, studies revealed that miRNA could be used as diagnostic and prognostic biomarkers for patient stratification and also as therapeutic targets, such as miR-215, miR-299-5p, miR-411 and miR-452, which were selected as potential biomarkers for breast cancer detection [7].

To find cancer-related miRNAs, microarray data analysis is a useful method to analyze the difference between miRNA expression of normal tissues and those of tumor tissues. Several approaches have been established to investigate the relationship between miRNA expression profiles of normal tissues and those of tumor tissues [8,9].

In addition to using microarray expression to find miRNA biomarkers for cancer, in this study, we propose a method, using miRNA stem-loop sequence structure, to help improve miRNA biomarker prediction. From a biological perspective, it is reasonable to assume that miRNAs with a similar phylogenetic structure are more likely to regulate the same disease. Nucleotide substitution models and the phylogenetic tree are widely-used tools to classify DNA or RNA sequences and to explore the level of sequence similarity [10,11,12,13]. In this study, we adopt a phylogenetic tree to present the similarity level of different miRNA sequences. As a result, a method combining microarray analysis and phylogenetic trees is proposed to predict high-confidence miRNA biomarkers for cancers.

## 2. Results

We first introduce the data used in this study. Huang *et al.* (2007) published two datasets of miRNA and mRNA microarray expression profiles across 88 normal or cancerous tissue samples [14]. One is the expression profiles for 114 human miRNAs, and the other one is the expression profiles for 16,063 mRNAs. To discover miRNA target genes based on these datasets, the relative r-squared method (RRSM), was established to select high-confidence miRNA target genes [15,16,17]. This method is to adopt a regression model under a relative r-squared criterion to find the relationship between miRNA and miRNA targets, which is based on a relative instead of an absolute statistical point of view. To perform the RRSM, two thresholds for the *p*-value and the relative r-squared value need to be set in the RRSM. For more details of the method, refer to the website http://www.stat.nctu.edu.tw/hwang/website_wang%20new.htm. The data and RRSM codes can be also accessed on this website. In addition, an approach investigating miRNA-target interactions and tissue specificity through microarray data was proposed, and tissue specificity results are provided through a study based on these datasets [8].

Since the samples in these datasets involve tumor tissues, Wang (2014) adopted the RRSM approach to discover potential miRNA biomarkers of cancers based on one of these datasets. More details about adopting the datasets and methods in Wang (2014) are given in the Materials and Methods section. In this study, we propose using a phylogenetic tree method to classify these selected miRNA biomarkers [9] based on their stem-loop sequences. As a result, high-confidence miRNA biomarkers are selected using both the microarray data and sequence structure.

**Figure 1:**
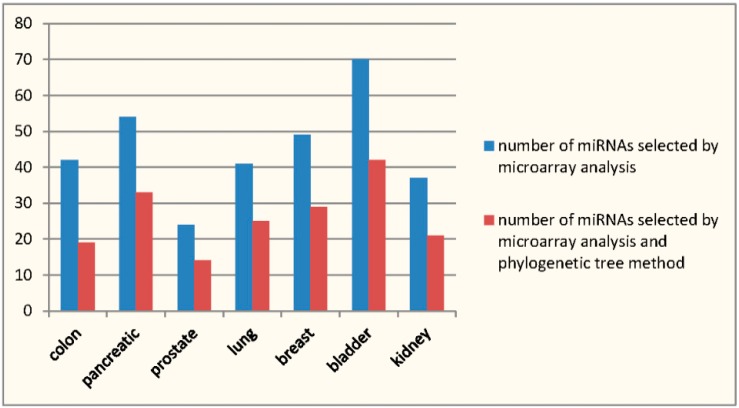
The numbers of miRNA selected by the proposed method or the microarray analysis.

There are a total of 53 miRNAs that have been selected by the proposed method. The steps of implementing this method are given in the Materials and Methods section. The 53 miRNAs are classified into 28 sets according to this method by combining both the RRSM and the phylogenetic tree criteria. The results are presented in Table 1, which lists the selected miRNAs and their cancer targets. Figure 1 shows the numbers of miRNAs selected by the proposed method or the RRSM.

The miRNAs in each set have the potential to be involved in some particular cancers’ development. In these 28 sets, there are three sets of miRNAs discovered for the single bladder cancer. There are eight miRNAs in these three sets, including miR-17, miR-20, miR-106a, miR-106b miR-92, miR-25, miR-16 and miR-195. The reason that these eight miRNAs are separated into three sets is that they are clustered into three different branches in the phylogenetic tree. For miR-92, since it was recently described as a family, including miR-92a and miR-92b, in the phylogenetic analysis, the sequence used for miR-92 is based on the stem-loop sequence of hsa-mir-92a-1 accessed from miRBase [18,19]. The miRNAs in the same set have a more similar phylogenetic structure than the miRNAs, which are not in the same set. There is also one set of miRNAs discovered for the single pancreatic cancer, which only includes miR-143.

In addition to the bladder cancer and pancreatic cancer, we do not find other miRNAs that are only related to a single cancer in these seven cancers through the proposed method. The other selected miRNAs are related to more than one cancer. The two sets of miRNAs, including miR-125a, miR-125b, miR-200b, miR-23a and miR-23b, are involved all seven cancers. The next are miR-27a and miR-27b, which involve six cancers. The result reveals that bladder cancer involves more miRNAs than other cancers investigated in this study.

Part of the results in Table 1 can be validated by previous studies. The last column in Table 1 lists the studies related to the selected miRNAs and their target cancers. These studies, which show the strong relationship between these miRNAs and their target cancers either in a direct or an indirect way, can enhance the reliability of the proposed method.

The selected 53 miRNAs, which are chosen from both the phylogenetic structure and the microarray data analysis aspects, can be used as high-confidence biomarkers of these seven investigated cancers for further experiment validation. In addition, when an miRNA only involves one cancer’s development, this miRNA can be more useful as a biomarker of this cancer than other miRNAs that involve more than one cancer’s development, because we can more easily identify its cancer target. miR-17, miR-20, miR-106a, miR-106b, miR-92, miR-25, miR-16, miR-195 and miR-143 are selected to involve a single cancer’s development. They have potential to be useful miRNA biomarkers when the result can be confirmed by experiments.

## 3. Discussion

To provide a more confident validation of the selected results, we use the Human MicroRNA Disease Database (HMDD) [73] to demonstrate the advantage of the proposed method. The database HMDD provides experiment-supported evidence for human microRNA (miRNA) and disease associations, which is manually collected from publications [73]. To verify the adequacy of the method, we adopt the sensitivity and specificity criteria on HMDD to show that the results selected in this study are more accurate than the results selected by the method of only using the microarray analysis. The sensitivity measures the proportion of actual positives that are correctly identified as such, and the specificity measures the proportion of negatives that are correctly identified as such [74]. That is, the sensitivity is the ratio of the number miRNAs that are selected by the method and confirmed by HMDD to the number of miRNAs selected by the method; the specificity is the ratio of the number of miRNAs that are selected by the method, but not confirmed by HMDD, to the number of miRNAs that are not confirmed by HMDD.

The sensitivities and specificities of the proposed method for the seven cancers are presented in Table 2. Most of them are greater than 0.5 for six cancers, except the colon cancer, with a sensitivity 0.476 and a specificity 0.429, and the breast cancer with a specificity 0.429. Compared to the results presented in [9], which only adopted the microarray analysis, but were not associated with the phylogenetic tree method, the proposed method can increase both sensitivity and specificity. The results obtained by Procedure 1 in [9] have a sensitivity 0.405 and a specificity 0.347 for the colon cancer and a sensitivity 0.519 and a specificity 0.435 for the pancreatic cancer. In this study, we also use the data obtained by Procedure 1 in [9], but screen them again using the phylogenetic tree method. This shows that the microarray analysis combining the phylogenetic tree can increase the sensitivity from 0.405 to 0.476 and increase the specificity from 0.347 to 0.429 for colon cancer compared to the method only using microarray analysis. The sensitivity increase rate is (0.476 − 0.405)/0.405 = 17.28%, and the specificity increase rate is (0.429 − 0.347)/0.347 = 23.63%. For pancreatic cancer, it can increase the sensitivity from 0.519 to 0.586 and increase the specificity from 0.437 to 0.64, with a sensitivity increase rate (0.586 − 0.519)/0.519 = 12.9% and a specificity increase rate (0.64 − 0.437)/0.437 = 46.45%. All of these increase rates are greater than 10%, which shows the significant improvement of the proposed method. The sensitivities and specificities for the other five cancers were not presented in [9]. Therefore, we only compare the results for colon cancer and pancreatic cancer. The outcome reveals that the method combining the phylogenetic tree with the microarray analysis improves the method of only using the microarray analysis.

## 4. Materials and Methods

### 4.1. Microarray Approach

To predict target genes of miRNAs, many methods have been adopted to explore the relationship between miRNA expression microarray data and gene expression microarray data. One of the methods is the RRSM procedure, which is based on the regression model to discover high-confidence target genes for miRNAs [15,16,17]. This method has also been adopted to discover high-confidence miRNA biomarkers for cancers using the above-mentioned dataset [9]. We briefly describe the approach of applying RRSM to select high-confidence miRNA biomarkers for cancers in [9]. To select high-confidence miRNAs that are associated with a cancer’s development, first, we can apply the RRSM to find miRNA biomarkers using expression profiles in tumor tissues. To compare the expression profiles in tumor tissue to those in normal tissues, we can also apply the RRSM to select miRNA biomarkers in normal tissue. After obtaining miRNA biomarkers in tumor tissue and miRNA biomarkers in normal tissue, respectively, the biomarkers, which are selected using the tumor tissue, but are not selected using the normal tissue, are regarded as high-confidence miRNA biomarkers for a cancer.

Cancer-related miRNAs for seven cancers, including colon, prostate, pancreatic, lung, breast, bladder and kidney cancers, were revealed [9], which are summarized in Table 3. For example, miRNA-199b is related to kidney cancer, and miRNA-16 is related to bladder cancer. According to the miRNAs’ cancer targets in Table 3, we classify the miRNAs that are related to same cancers into the same group.

**Figure 2:**
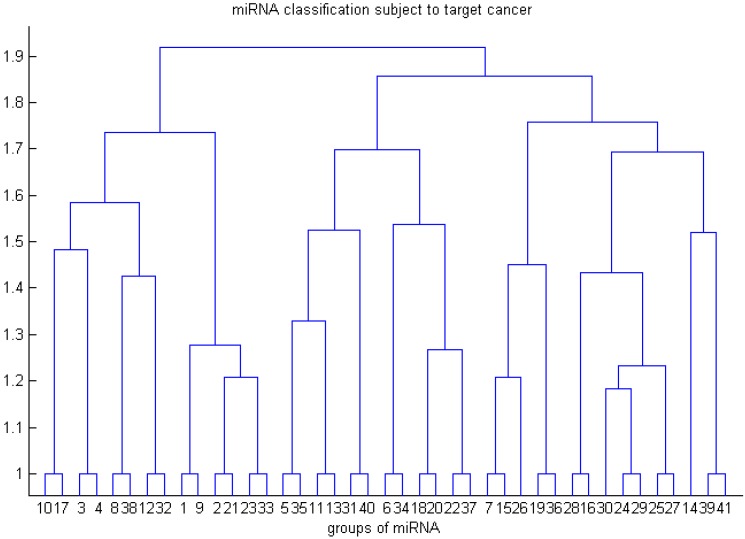
Ninety miRNA classifications subject to target cancer.

To classify the miRNAs subject to the results in Table 3, we applied a hierarchical tree method to group the results in Table 3 and explore the relationship between these groups. The results in Table 3 can be classified into 41 groups. The group number of an miRNA in Table 3 indicates that this miRNA belongs to the group corresponding to this group number in Figure 2. For example, Branch 1 of Figure 2 constitutes 4 miRNAs (miR-124a, miR-9, miR-182, miR-135); Branch 2 constitutes 6 miRNAs (miR-125b, miR-23b, miR-23a, miR-125a, miR-200b, miR-200c); Branch 3 constitutes 1 miRNA (miR-146). Figure 2 shows the clustering relationship between these 41 groups.

About the details of plotting the tree in Figure 2, for an miRNA, we need to assign a vector corresponding to this miRNA subject to its cancer targets listed in Table 3. For example, the vectors corresponding to miR-124a and miR-146 are (1, 1, 1, 1, 1, 1, 0) and (1, 0, 0, 1, 0, 1, 1), respectively. The steps of constructing a hierarchical tree method are first to calculate the pairwise distances for each pair of the 90 vectors and then to adopt a clustering approach to classify the miRNAs using the calculated pairwise distances. We adopt the Euclidean distance to calculate the pairwise distance between vectors. For the clustering approach used in the second step, we apply the unweighted pair-group method with arithmetic averages (UPGMA) to plot the tree. The MATLAB codes for plotting this tree are:

**Y = pdist (data); Z = linkage (Y, ‘average’); dendrogram (Z)**

where “**data**” is a 90 × 7 matrix obtained from Table 3. The hierarchical tree of miRNAs subject to their cancer targets is plotted in Figure 2.

The 90 miRNAs are classified into 41 groups. The last column of Table 3 shows the group numbers of these miRNAs.

### 4.2. Phylogenetic Tree

A phylogenetic tree is a widely-used tool for investigating evolutionary relationship between DNA sequences. In this study, we adopt the phylogenetic tree approach to cluster miRNAs in terms of their phylogenetic structure. The phylogenetic analysis cannot be used to analyze microarray data alone, because it can only be adopted to analyze sequence data. However, it can be used as an ancillary tool to find high-confidence miRNA biomarkers by combining it with a microarray analysis. To access miRNA sequences, miRBase is a useful database that provides most discovered miRNA sequences for many species [18,19]. In this study, the miRNA stem-loop sequences are accessed from miRBase and are used to build a phylogenetic tree. For example, the accession number of Homo sapiens miR-211 (has-mir-211) is MI0000287, and its stem-loop sequence is UCACCUGGCCAUGUGACUUGUGGGCUUCCCUUUGUCAUCCUUCGCCUAGGGCUCUGAGCAGGGCAGGGACAGCAAAGGGGUGCUCAGUUGUCACUUCCCACAGCACGGAG.

The stem-loop sequence of a precursor miRNA includes of a 5p mature miRNA sequence and a 3p mature miRNA sequence. It can provide more information of a miRNA than only using a mature miRNA sequence.

To plot the phylogenetic tree of these 90 miRNAs, we need to adopt a substitution model to calculate pairwise distances between sequences and adopt a hierarchical clustering distance method to build a tree. In this study, we first calculate the pairwise distances between sequences using the Jukes–Cantor substitution model method. After that, we use the nearest distance method (single linkage method) to plot the phylogenetic tree. The MATLAB codes for plotting the phylogenetic tree are:

**distances = seqpdist (seqs, ‘Alphabet’, ‘NT’)**

**Figure 3:**
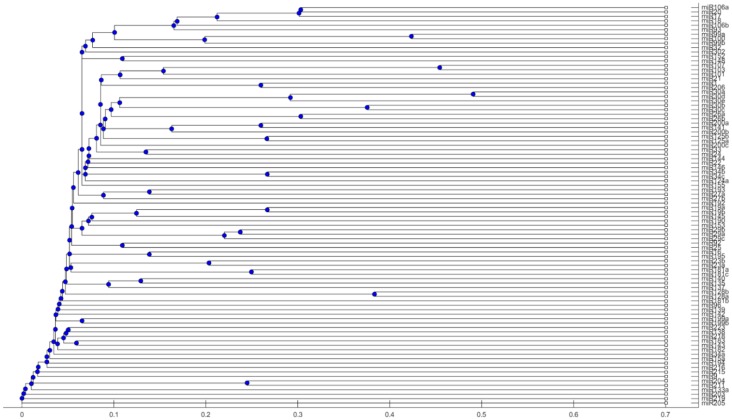
Phylogenetic tree of 90 miRNAs.

**phylotree = seqlinkage (distances, ‘single’, seqs)**

where **seqs** is the 90 miRNA stem-loop sequences. The phylogenetic tree of miRNAs subject to their stem-loop sequences is plotted in Figure 3.

The miRNAs in the same clade are considered to have a similar phylogenetic structure. For example, in Figure 3, miR-30a, miR-30b and miR-30e are in a clade, and miR-30a and miR-30d are in a nested clade.

### 4.3. Procedure of Discovering High-Confidence miRNA Biomarkers

In order to select high-confidence miRNA biomarkers for cancers, we propose a method combining the results of cancer target prediction and the phylogenetic tree. The first step of the method is to find miRNAs that are classified in the same sub-branch in Figure 3. For example, the 5 miRNAs, miR-30a, miR-30b, miR-30c, miR-30d and miR-30e, are in a clade. From Figure 2, these 5 miRNAs are also classified into the same group according to cancer target prediction. From both results, we have more confidence to believe that these 5 miRNAs are involved in pathological mechanism of some particular cancers. Combining the above two methods, we develop a method to classify miRNAs subject to cancer development. The steps of performing this method are given as follows.

Procedure of the proposed method:

**Step** **1.** Use the stem-loop sequences of miRNAs to build a phylogenetic tree.
**Step** **2.** Use RRSM or other microarray data analysis to select cancer-related miRNA. Cluster miRNAs into different groups subject to the cancer target prediction result; see Table 3.
**Step** **3.** Collect miRNAs in the same clade in the phylogenetic tree of **Step 1**. If miRNAs in the same clade belong to the same group of miRNAs that are clustered in **Step 2**, these miRNAs are selected to be high-confidence miRNA biomarkers for particular cancers.

**Figure 4:**
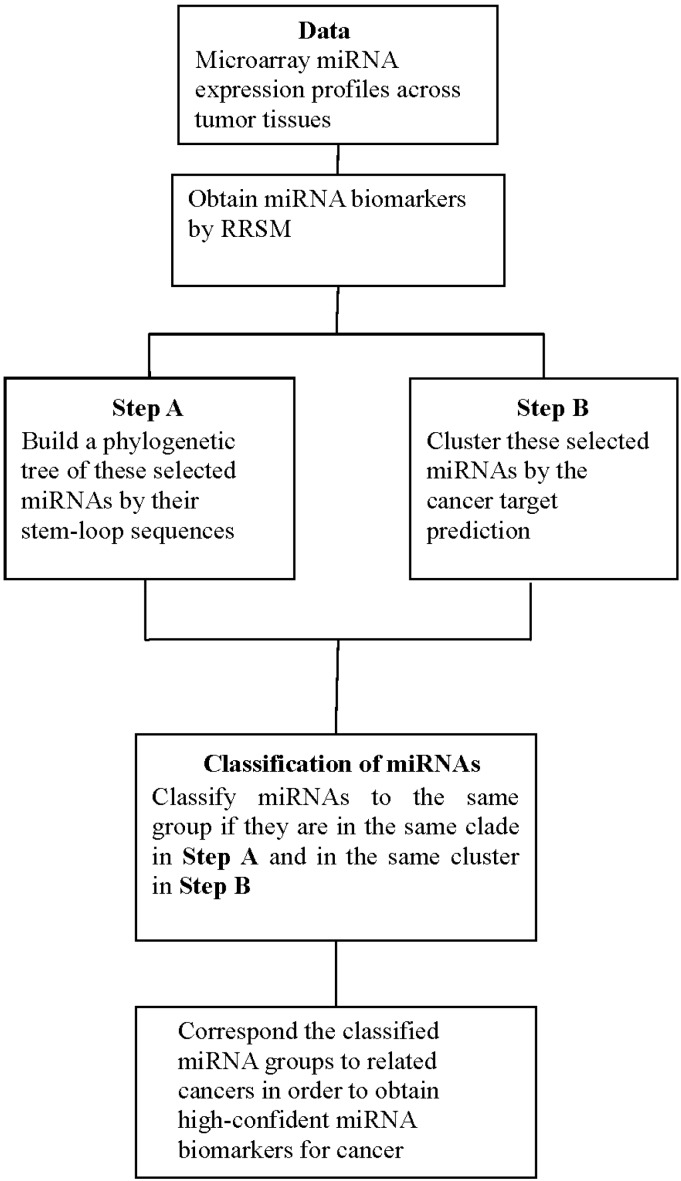
The flowchart of the method.

Although the steps in this procedure are illustrated using the cancer-related miRNAs, this approach can be generally used to find miRNA biomarkers for other diseases. A flowchart of the method is provided in Figure 4.

## 5. Conclusions

In this study, we propose combining the phylogenetic tree analysis with the microarray analysis to increase the accuracy of miRNA biomarkers’ prediction. To analyze microarray data, the phylogenetic analysis cannot be used alone without combining a microarray analysis because the phylogenetic tree is used to cluster sequences, but not microarray data. Although the phylogenetic analysis cannot directly extract information from microarray data, the results in this study show that it can be a useful ancillary tool to select high-confidence miRNA biomarkers by combining it with a microarray analysis.

## Figures and Tables

**Table 1:** The classification of miRNAs that are selected by the proposed method subject to cancer development.

MircoRNA	Cancer	Validation from Other References
miR-17, miR-20, miR-106a, miR-106b	Bladder	[20,21]
miR-92, miR-25	Bladder	[22,23]
miR-16, miR-195	Bladder	[24,25]
miR-143	Pancreatic	[26,27]
miR-99a, miR-99b, miR-100	Pancreatic, Lung	[28,29]
miR-103, miR-107	Pancreatic, Lung	[30,31]
miR-193	Colon, Breast	[32,33]
miR-205	Pancreatic, Bladder	[34,35]
miR-139	Colon, Breast, Bladder	[34,36]
miR-133a	Colon, Pancreatic, Bladder	[37,38]
miR-152, miR-148	Lung, Breast, Bladder	[39,40]
miR-26a, miR-26b	Pancreatic, Breast, Bladder	[39,41]
miR-30a, miR-30b, miR-30c, miR-30d, miR-30e	Colon, Pancreatic, Lung, Bladder	[42,43]
miR-34b, miR-34c	Colon, Lung, Breast, Kidney	[44,45]
miR-19a, miR-19b	Colon, Lung, Breast, Bladder	[46,47]
miR-101	Colon, Pancreatic, Breast, Kidney	[48,49]
miR-146	Colon, Lung, Bladder, Kidney	[48,50]
miR-137	Colon, Pancreatic, Bladder, Kidney	[51,52]
miR-194	Prostate, Pancreatic, Lung, Bladder	[53,54]
miR-29a, miR-29b, miR-29c	Prostate, Breast, Bladder, Kidney	[55,56]
miR-181a, miR-181c	Pancreatic, Breast, Bladder, Kidney	[57,58]
miR-128a, miR-128b	Prostate, Pancreatic, Breast, Bladder	[59,60]
miR-204, miR-211	Pancreatic, Breast, Bladder, Kidney	[61,62]
miR-145	Colon, Pancreatic, Breast, Bladder, Kidney	[63,64]
miR-96	Colon, Prostate, Pancreatic, Breast, Kidney	[65,66]
miR-27a, miR-27b	Prostate, Pancreatic, Lung, Breast, Bladder, Kidney	[67,68]
miR-125a, miR-125b, miR-200b	Colon, Prostate, Pancreatic, Lung, Breast, Bladder, Kidney	[69,70]
miR-23a, miR-23b	Colon, Prostate, Pancreatic, Lung, Breast, Bladder, Kidney	[71,72]

**Table 2:** Numbers of miRNA and the sensitivities and specificities of the proposed method. HMDD, Human MicroRNA Disease Database.

	miRNAs Selected in [9]	miRNAs Confirmed by HMDD	miRNAs Selected by the Method	miRNAs Selected by the Method and Confirmed by HMDD	Sensitivity	Specificity
	A	B	C	D	D/B	(C − D)/(A − B)
Colon	42	21	19	10	0.476	0.429
Pancreatic	54	29	33	17	0.586	0.64
Prostate	24	2	14	1	0.5	0.591
Lung	41	34	25	20	0.588	0.714
Breast	49	42	29	26	0.619	0.429
Bladder	70	34	42	17	0.5	0.694
Kidney	37	10	21	6	0.6	0.556

**Table 3:** Potential cancer-related miRNAs marked by “v”.

	Colon	Prostate	Pancreatic	Lung	Breast	Bladder	Kidney	Group Number in Figure 2
miR-124a	v	v	v	v	v	v		1
miR-9	v	v	v	v	v	v		1
miR-182	v	v	v	v	v	v		1
miR-135	v	v	v	v	v	v		1
miR-125b	v	v	v	v	v	v	v	2
miR-23b	v	v	v	v	v	v	v	2
miR-23a	v	v	v	v	v	v	v	2
miR-125a	v	v	v	v	v	v	v	2
miR-200b	v	v	v	v	v	v	v	2
miR-200c	v	v	v	v	v	v	v	2
miR-146	v			v		v	v	3
miR-199	v	v		v		v	v	4
miR-1	v		v					5
miR-30b	v		v	v		v		6
miR-200a	v		v	v		v		6
miR-30a	v		v	v		v		6
miR-30d	v		v	v		v		6
miR-30c	v		v	v		v		6
miR-30e	v		v	v		v		6
miR-137	v		v			v	v	7
miR-19a	v			v	v	v		8
miR-19b	v			v	v	v		8
miR-203	v		v	v	v	v		9
miR-155	v		v	v	v	v		9
miR-33	v			v	v	v	v	10
miR-219	v							11
miR-216	v							11
miR-223	v				v	v	v	12
miR-193	v				v			13
miR-29b		v			v	v	v	14
miR-29c		v			v	v	v	14
miR-29a		v			v	v	v	14
miR-206	v		v				v	15
miR-218		v	v		v	v		16
miR-128b		v	v		v	v		16
miR-128a		v	v		v	v		16
miR-34a	v			v	v		v	17
miR-34b	v			v	v		v	17
miR-34c	v			v	v		v	17
miR-194		v	v	v		v		18
miR-138		v	v	v				19
miR-96	v	v	v		v		v	20
miR-27b		v	v	v	v	v	v	21
miR-27a		v	v	v	v	v	v	21
miR-99a			v	v				22
miR-100			v	v				22
miR-107			v	v				22
miR-103			v	v				22
miR-99b			v	v				22
miR-181a			v		v	v	v	23
miR-181b			v		v	v	v	23
miR-204			v		v	v	v	23
miR-211			v		v	v	v	23
miR-181c			v		v	v	v	23
miR-24	v		v	v	v	v	v	24
miR-205			v			v		25
miR-215			v			v	v	26
miR-192			v			v	v	26
miR-21	v	v	v			v	v	27
miR-190			v		v	v		28
miR-26a			v		v	v		28
miR-144			v		v	v		28
miR-26b			v		v	v		28
miR-183	v	v	v		v	v		29
miR-22			v	v	v	v	v	30
miR-145	v		v		v	v	v	31
miR-140	v					v		32
miR-139	v				v	v		33
miR-143			v					34
miR-133a	v		v			v		35
miR-18				v				36
miR-101	v		v		v		v	37
miR-152				v	v	v		38
miR-148				v	v	v		38
miR-141				v	v	v		38
miR-302					v		v	39
miR-93					v		v	39
miR-16						v		40
miR-92						v		40
miR-142						v		40
miR106a						v		40
miR-17						v		40
miR-195						v		40
miR-20						v		40
miR-15a						v		40
miR-153						v		40
miR-106b						v		40
miR-25						v		40
miR-32						v		40
miR-199b							v	41
